# Indigenous wood species classification using a multi-stage deep learning with grad-CAM explainability and an ensemble technique for Northern Bangladesh

**DOI:** 10.1371/journal.pone.0328102

**Published:** 2025-07-29

**Authors:** Ashrafun Zannat, Md. Saiful Islam, Md. Shahriar Zaman, Sadman Saif

**Affiliations:** 1 Institute of Information and Communication Engineering, Bangladesh University of Engineering and Technology, Dhaka, Bangladesh; 2 Department of Computer Science and Engineering, Bangladesh Army University of Science and Technology, Nilphamari, Bangladesh; South China University of Technology, CHINA

## Abstract

Wood species recognition has recently emerged as a vital field in the realm of forestry and ecological conservation. Early studies in this domain have offered various methods for classifying distinct wood species found worldwide using data collected from a particular region. An image dataset has been developed for wood species classification of Bangladeshi forest. Our aim is to address the gaps by comparing and contrasting our developed sequential Convolutional Neural Network based BdWood model with several deep learning, ensemble technique, Machine learning classification models on specific wood species identification for Bangladeshi forests. Using our own dataset, comprising more than 7119 high-quality captured images representing seven types of wood species of Bangladesh. It is found that DenseNet121 is the clear winner in our thorough evaluation among seven pre-trained models. The highest accuracy of DenseNet121 is achecived 97.09%. In addition, our customized BdWood model, which is adapted to the desired outcome, produced results that are excellent. BdWood model achieves a training accuracy of 99.80%, validation accuracy of 97.93%, an F1-score of 97.94%, and an outstanding ROC-AUC of 99.85%, demonstrating its effectiveness in wood species classification. Gradient-weighted Class Activation Mapping (Grad CAM) is used to interpret the model’s predictions, providing insights into the features contributing to the classification decisions. Finally, to make our research practically applicable, we have also developed an Android application as a tangible outcome of this work.

## Introduction

Due to the major biodiversity of species, most dataset are not available for indigenous species to a particular country or specific climatic zone [1–3]. Visual inspection of physical and anatomical features is an important identification marker for conventional wood identification. Wood classification is required to observe the physical features such as color, figure, and luster, as well as anatomical structure like size, vessels, axial parenchyma cells, and rays [4]. Visual examination of wood species in general person can result an erroneous selection of wood species. A wood analyzer can give an approval for appropriate wood species, that can create a customize wood species classification model. While these databases have simplified the identification of rare woods, traditional visual inspection remains preferred for the efficient identification of commercial woods. Early computer-aided wood identification systems relied on explicit programming, requiring users to manually input all identification rules, which proved inefficient given the complexity and diversity of wood characteristics. This programmatic approach also hindered the global adoption of such systems. Computer based wood identification evolved, incorporating web-based resources like ‘Inside Wood’ at North Carolina State University and ‘Microscopic Identification of Japanese Woods’ at the Forestry and Forest Products Research Institute (FFPRI) in Japan are valuable and cover a broad range of wood species, they still require expert knowledge of wood anatomy. The challenges associated with traditional computer-aided identification systems have opened the door for advancements in machine learning (ML) to enhance the process.

A branch of artificial intelligence (AI) is Machine learning, a system can learn to determine the appropriate actions solely from inputting data using pre-trained algorithms, without requiring explicit instructions from humans. In an ML model, users no longer need to provide the rules for identifying wood, nor do wood anatomists that can need to identify the critical features for wood identification. A technology based intelligence system can enables computers to detect information from images and extract significant features plays an important role in this process. When CV and ML are combined for automated wood identification, this approach is referred to a computer vision-based wood identification [5, 6]. In AI systems that integrate CNN and ML have been making significant advancements in general image classification, and this progress extends to wood identification, where related studies have been on the rise.

It is impossible to train enough field identification workers to correctly identify the timber and screening of fraudulent species. [7, 8] meet the growing demands. However, wood species identification requires some anatomy knowledge and extensive experience, and also required some money and time [9]. Different approaches have been proposed, including mass spectrometry, near-infrared spectroscopy [10, 11], stable isotopes [12, 13], and DNA-based methods [14, 15]. With high costs and procedural complexities these methods can’t proposed a practical tools for visual inspections. In this circumstance, computer vision (CV)-based identification techniques and machine learning (ML) models pay a vital role in wood automation. significant. Automated wood identification systems have a promising solution in our environment and industries. Several techniques used in several areas,like wood grading, quality evaluation, and defect detection. In our aim is to create a CV-based identification procedures and machine learning (ML) models that can highlight key feature of the wood species, and introduce emerging interests in CV-based wood anatomy. Class Activation Mapping (CAM) is applied for identifying important regions in an image that are required for a CNN’s classification decision. However, Generalization of CAM is used in more complex models like fully connected CNN models, models used for structured outputs, and models used for tasks involving multiple inputs without required any architectural changes. However, CAM is limited to CNNs without fully connected layers, which may lead to reduced model complexity and performance in exchange for greater transparency.

Previous research on wood species classification has largely relied on datasets from other regions. In previous contrast between machine learning and CNN techniques, CNNs have demonstrated remarkable success in image classification proposed by M.M Hossain *et al*. [16]. As we see the vast biodiversity of wood, It is a most important task to opt a databases that can cover the native species to a country or a specific climatic region [1]. Used stereo type of image for wood classification and performed machine learning algorithms. SVM has been performed more accurately than k-NN for wood identification. This model achieved a less of 87.7%. [17] with 481 number of images. For the wood identification, [18] they used 128-dimensional SIFT for extracting features from Fagaceae micrographs to 17 dimensions, using the reduced feature set was quite accurate.

Observing various microscopically anatomical features, wood identification is performed in the laboratory [19], collecting such core images is challenging and costly. Tang *et al*. [20] exposed a smart portable device for macroscopic wood image identification that was a phone-based device. Different wood images are used wood classification. Macroscopic images are the most commonly used image types.Used 2942 macroscopic images and applied CNN achieved 95.8%. [21] A deep transfer learning approach with pre-trained Convolutional Neural Network (CNN) models such as ResNet-50 and InceptionV3 achieved a classification accuracy of 95.88% using the “WOOD-AUTH" dataset proposed by Kirbas and Cifci [22]. In Table 1, it is clear that various researchers have experimented with a wide range of classification models across several wood species datasets, including macroscopic image dataset from “WOOD-AUTH” dataset and other regions dataset. These studies have produced a broad range of accuracy scores, with most datasets achieving impressive results between 91% and 98%. However, macroscopic image dataset from “WOOD-AUTH” [22] dataset and other regions dataset are not required for our country aspect. As a result, our research is dedicated to creating a Bangladeshi wood species dataset and improving the classification accuracy to align with the higher performance standards observed in other datasets. Amri *et al*. [23] focuses on hybrid architectures similar to our approach (e.g., combining MobileNet, Inception, and VGG) and employ SHAP (SHapley Additive exPlanations) to provide interpretability, making our model more transparent and reliable. Which includes ten plant species specific to Saudi Arabia, addressing a gap in region-specific datasets. Gulzar *et al*. [24] for soybean classification, they customized the InceptionV3 architecture with different layers.They achieved 98.73% accuracy.

**Table 1:** Conducted research on wood science.

Paper	Limitations	Classifiers	Test accuracy
[25]	Used Brazilian wood species dataset.	4 CNN Classifier	Highest accuracy DenseNet 98.13%
[26]	Used small dataset which contain only 1000 image of Indian wood	The Grey-Level Co-occurrence Matrix (GLCM) is used as a texture classification technique.	The recognition rate up to 95%.
[27]	Used macroscopic image dataset from “WOOD-AUTH” dataset.	ResNet50, InceptionV3, Xception, and VGG19	Classification accuracy of 95.88%
[28]	Less accuracy	Support Vector Machines (SVM)	Classification rate of 91.47% and 84.2%.
[29]	Difficult to collect wood core image.	Used wood species identification with CNN models	93% wood image patches and 98.7% of wood core images.

Grad-CAM is used to identify the wood region that pay a vital role in our classification models for classify our seven species correctly. Our model shown a great accuracy in our given datasets. Ensemble is a troupe learning technique used to design a improved version of machine learning. It reduces variance, helps prevent overfitting, making model more robust to noise and fluctuations in the data. Models built through bagging are more stable and less prone to overfitting. Since each model is trained independently, bagging can be parallelized, speeding up the training process.

In this paper, we have tackled the aforementioned challenges and made the following key contributions:

- Developed a brand new indigenous wood species image datasets for northern Bangladesh are a vital challenge for us. The models are being trained on fresh data that hasn’t been seen by the models before.
- The pre-trained models (DenseNet121, Xception, ResNet152V2, InceptionV3, VGG16, VGG19, MobileNetV2) apply on the newly collected data.
- The models have evaluated ROC-AUC curve.The AUC helps determine how well the models differentiate between classes, providing a robust measure of performance.
- Traditional KNN and SVM models are used for comparing deep learning models are more applicable for image data classification.
- Building an ensemble method that reduces variance by training multiple models on samples and aggregating their predictions and a comparative analysis with a customized Sequential CNN model for classification of indigenous wood species in Bangladesh.
- Our research devised to the field of model explain ability by employing Grad-CAM (Gradient-weighted Class Activation Mapping) in models applied Grad-CAM in our novel approach using sequential Convolution Neural Network.
- Finally, we developed an Android application as part of this research, offering a practical contribution to the field.

This paper is organized as follows: the design methodology with experimental setup is explained in the Materials and Methods section. The experimental findings and an analysis of the overall outcomes of our proposed approach presents in the result and discussion section. Also, the models comparison is shown in the result section. Lastly the conclusion section summarizes the paper and outlines directions for future work.

## Materials and methods

**Fig 1:**
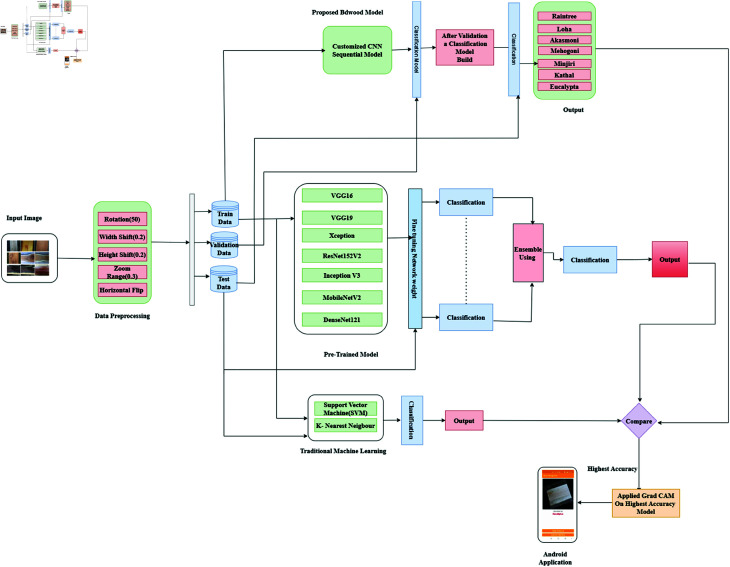
Recommended methodology for wood species classification and build an android application.

In Fig 1, this framework depicts a detailed explanation of the proposed scheme. This research divided into five major parts: i) Dataset development. ii) Data preprocessing. iii) Model building using CNN pre-trained and Machine Learning models. iv) Create an ensemble of heterogeneous models v) Model explainability, and vi) Developed an Android application.

### Dataset development

In Bangladesh according to BFD 2010 [30], 84% area has been classified as natural forest and 16 % area as plantation forest. There are three types of forest we have considered, those are evergreen/semievergreen, moist/dry deciduous and swamp forests. Dinajpur, Rangpur and Thakurgaon districts are the north part of the country. The moist/dry deciduous forests are distributed at the north part of the country [30] We have visited various wood mills and wood shops in Dinajpur and Rangpur, Bangladesh collected pictures of seven types of wood with the help of skilled people who knows wood well. We have collected more than 2000 jpg image that represented seven distinct wood species: Akashmoni, Mehogoni, Eucalyptus, Loha, Kathal, Minjiri, and Raintree. In the image acquisition process, most of the studies are used cross-sectional images of wood blocks, although some studies may in- clude images of lumber surfaces or three orthogonal sections [27]. To reveal the anatomical characteristics of the wood, the surfaces of the blocks are often cut with a knife or sanded with sandpapers. Using a digital camera or stereo microscope, Macroscale images are captured directly from the wood blocks. A standard procedures such as softening, cutting, staining, dehydration, and mounting are required for capturing microscale images. The quality of the images are varied depending on the lighting conditions. To ensure consistency, imaging modules equipped with optical systems are often used to control lighting uniformly [28, 29]. Additionally, image processing techniques such as filtering are applied to normalize the brightness of the captured images. Dataset and code can be accessed on GitHub at https://github.com/ashrafunzannat/Wood_Classification.

#### Image type.

Various image types are used for wood identification, each offering unique insights into wood characteristics.Macroscopic images are used commonly. X-ray computed tomographic (CT) images, stereograms, and micrographs are also used for wood identification. Magnification are not required for Macroscopic images, using a regular digital camera and highlight large wood cells and aggregates such as annual rings, rays, and vessels [31, 32]. Due to the ease of acquisition by simply smoothing the wood surface, these images are preferred for field-deployable systems [29, 33]. X-ray CT images, which are slices generated by X-ray CT scans, provide a non-destructive means to observe large wood cells and aggregates, making them ideal for identifying wooden objects with limited sampling, such as cultural properties.

In our collected dataset consists of images, these are JPG (Joint Photographic Experts Group) format. JPG is a widely-used raster image with its lossy compression characteristics. JPG format is specially helped us for image analysis with low cost, easy to collect and reducing file size while maintaining a reasonable level of image quality. It is suitable for our application with high-resolution information and also required for wood species classification and detection. The reliability of our models and to boost the variety of the dataset, different smartphone such as the Samsung A50, Redmi 10 2022, and Redmi 5 Plus—were used to shoot the photographs. Here, we find the dataset validation form completed by the expert at the following link: https://github.com/ashrafunzannat/Wood_Classification. This form includes a detailed review and validation of the dataset for the research.

### Data preprocessing

Pre processing is an important part for building a models. The goal is to standardize the input data so that the model can build efficiently from it. Load the image dataset using such as TensorFlow, Keras, PyTorch, or OpenCV libraries. Many CNNs models required some specific size (e.g., 224 x 224 for models)of images so that we need to resize the images using OpenCV libraries function cv2.resize(image, (224, 224)).Then normalization is used to scale the pixel values. Standardization of the dataset by subtracting the mean and dividing the standard deviation of the dataset.

#### Data augmentation.

Data augmentation is a used to artificially increase the size of dataset and this can help to reduce overfitting and improve generalization. Common augmentation techniques are applied for data processing such as

i) **Flipping:** Horizontally or vertically.
ii) **Rotation:** Small rotations, e.g., ±20 degrees.
iii) **Zooming:** Random zoom in/out.
iv) **Brightness adjustment:** Changing the brightness level.
v) **Shifting:** Slight translation of images.

**Fig 2:**
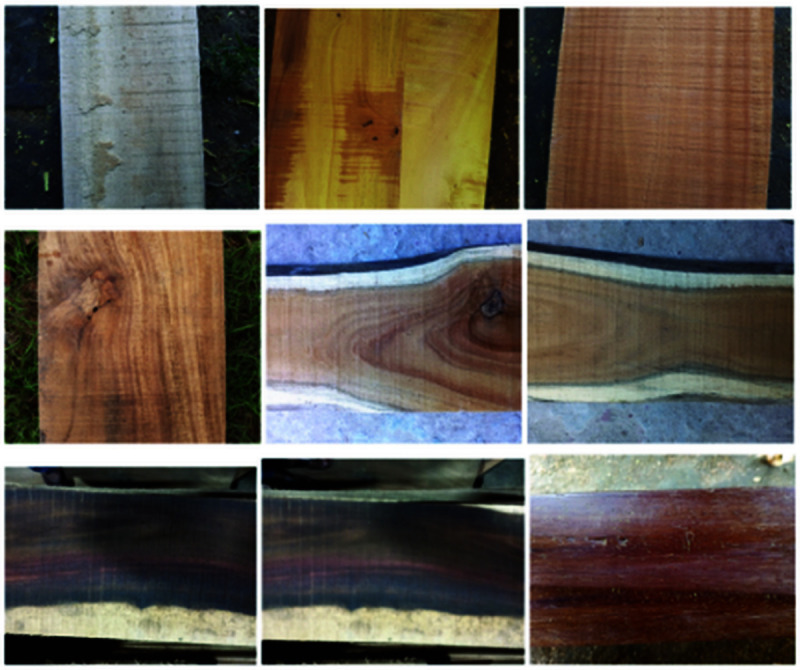
Before pre-processing image file.

**Fig 3:**
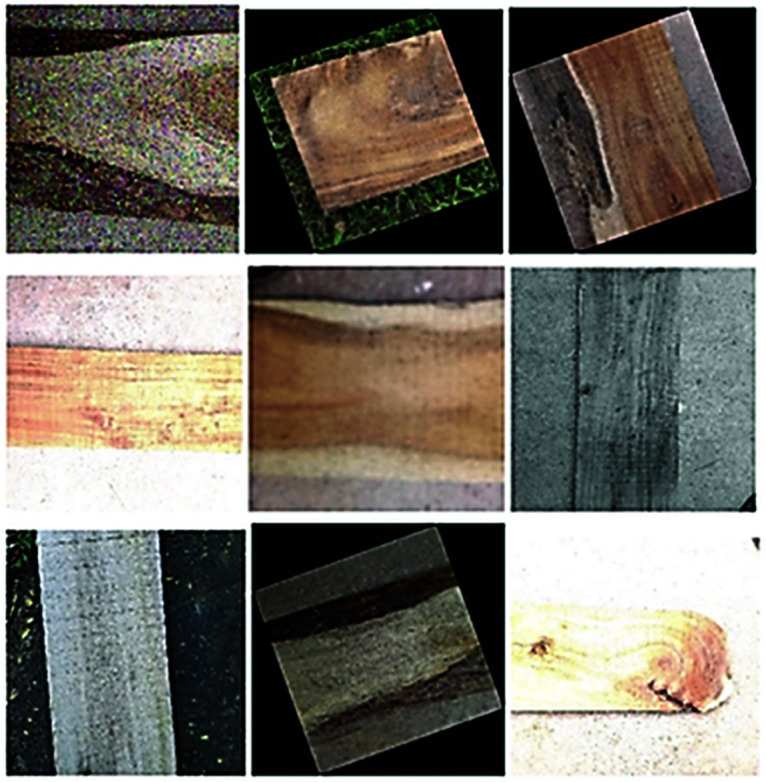
After pre-processing image file.

Fig 2 presents a few sample images of our dataset, whereas Fig 3 shows some images after applying data augmentation and also used those images for train our models.

### Model building using pretrained CNN and traditional ML classifiers

**VGG16** is widely used for simple and popular for its earlier architectures, which helps in capturing complex patterns in the data. Its architecture comprises a total 16 layers and all the convolution layers employ a compact filter with size of 3x3 with a stride of 1 and padding to preserve spatial resolution. Each convolutional layer is followed by a ReLU activation function and a softmax function is applied in the output layer for classification.

**VGG19** is an extension of the VGG family of deep convolutional neural networks, specifically designed for large-scale image classification tasks. It is an improvement over VGG16 by increasing the depth of the network to 19 layers, providing better feature extraction capabilities, but at the cost of increased computational complexity and more parameters.

**DenseNet121** is a unique connectivity pattern, every layer is connected to every other layer in a dense block. This allows each layer to access gradients more easily during back propagation and consists of 121 layers, with alternating convolution and max pooling layers.DenseNet121 has fewer parameters compared to architectures like VGG and ResNet because it doesn’t need to relearn redundant features [34].

**InceptionV3** is an improved version of inceptionV1 and V2. It’s perform convolutions with different filter sizes (1 x 1, 3 x 3, 5 x 5) in parallel and reduce computational complexity while maintaining high performance. nceptionV3 uses factorized convolutions to split large convolutions (e.g., 7 x 7) into smaller, more efficient operations (e.g., two 3 x 3 convolutions) and also add auxiliary classifiers at intermediate layers.It involves the utilization of binary cross-entropy loss function [35].

**Xception** is an extension of the Inception architecture. It is designed to utilizing the depth wise separable convolutions instead of the standard convolutions used in traditional architectures. It’s ability to learn highly discriminative feature with fewer parameters [36].

**MobileNetV2** is a lightweight architecture for mobile and embedded devices.MobileNetV2 uses inverted residuals with linear bottlenecks.MobileNetV2 process the data in a low dimensional space the expand it into a higher dimensional space. The use of depth wise separable convolutions and inverted residuals allows for fewer parameters and faster inference time compared to other networks like VGG or Inception.

**SVM** is performed for both linear and non-linear classifiers that can differentiate many types of object. SVM is used for the linear kernel. SVM tries to find a hyperplane (a decision boundary) that best separates the data points of different classes. Used a parametric learning type and expensive training for getting prediction is fast.

SVM allows for misclassifications by introducing slack variables and controlling the margin-error trade-off for noisy data [37].

**One-vs-Rest (OvR)** is used when the class number is not too large, and dataset is relatively balanced. It works well when the class number is relatively small and training resources are limited. This results in *K* SVMs if there are *K* classes in the dataset.

**One-vs-One** approach is typically used in image classification tasks when the class number is large, or when the dataset is well-balanced between classes. How it works: For every pair of classes, a separate binary SVM is trained. For *K* classes, this results in $\frac{K(K-1)}{2}$ classifiers. During prediction, each classifier votes for one of the two classes, and the class with the most votes is predicted.

**K-Nearest Neighbors (KNN)** is a traditional machine learning classification algorithm that works by assigning a label to an image based on the majority vote of its k-nearest neighbors in the feature space. Given a test image represented as a feature vector $x_{text}\in {\mathbb{R}}^{⋉}$. The algorithm compute *x*_*test*_ to all the training ${x^{i}}_{train}\in {\mathbb{R}}^{⋉}$ The most commonly used distance metric is the Euclidean distance:

$$d(x_{test},x_{train}^{i})=\sqrt{\sum_{j=1}^{n}(x_{test,j}-x_{train,j}^{i}{)}^{2}}$$ (1)

Once the distances are computed, the k closest training samples are selected, and each of these neighbors casts a vote for its class.

### Heterogeneous ensemble methods

A heterogeneous ensemble is an ensemble technique that can combine the predictions of multiple ML and several DL models of different types or architectures to achieve better performance than any individual model. An ensemble model can help capture various patterns and reduce the risk of overfitting or bias inherent to a single model.

Voting: For classification tasks, models may use majority voting or weighted voting to decide the final result of the prediction.

Averaging: For regression, the predictions are averaged to produce the final output.

Stacking: A meta-model learns to combine the predictions from individual models.

Reduces bias by incorporating models with different perspectives. Mitigates the weaknesses of individual models. Often achieving better generalization, especially with diverse and complex datasets.

**Model-specific predictions.** Each model *M*_*i*_ (i=1,2,…,n) generates predictions for *k* classes. The prediction for an input *x* by model *M*_*i*_ is:

$$P_{i}(x)=[p_{i1},p_{i2},\ldots ,p_{ik}]$$ (2)

where *p*_*ij*_ is the predicted probability for class *j* by model *M*_*i*_.

**Ensemble aggregation (majority voting).** The ensemble combines predictions by averaging the probabilities across all models:

$$\bar{P}(x)=\frac{1}{n}\sum_{i=1}^{n}P_{i}(x)$$ (3)

where:

$$\bar{P}(x)=[{\bar{p}}_{1},{\bar{p}}_{2},\ldots ,{\bar{p}}_{k}]$$ (4)

and

$${\bar{p}}_{j}=\frac{1}{n}\sum_{i=1}^{n}p_{ij}$$ (5)

is the mean probability for class *j*.

The predicted class $\hat{y}$ is determined by selecting the class with the highest averaged probability:

$$\hat{y}=\arg\max_{j}\bar{P}(x)$$ (6)

**Accuracy calculation.** Accuracy is computed as the percentage of correctly predicted labels compared to the true labels:

$$\text{Accuracy}=\frac{\text{No. of Correct Predictions}}{\text{Total No. of Predictions}}\times 100$$ (7)

Let $y_{\text{true}}$ and $y_{\text{pred}}$ be the true and predicted labels, respectively. Then:

$$\text{Accuracy}=\frac{1}{N}\sum_{i=1}^{N}⊮(y_{\text{true},i}=y_{\text{pred},i})\times 100$$ (8)

where:

- *N* is the total number of samples.
- ⊮(condition) is the indicator function, which equals 1 if the condition is true and 0 otherwise.

### Proposed multi stage Bdwood convolutional neural network

**Fig 4:**
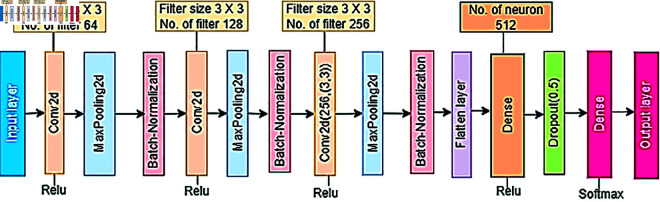
Methodology for a custom convolutional neural network BdWood model.

A Convolutional Neural Network (CNN) is used to build a block specially designed to recognize patterns in images. It is widely used for image classification. Fig 4 proposed the methodology for our customized multi-stage CNN model for indigenous Bangladeshi wood species. Moreover, it is called the BdWood model. This classification model can classify seven species with great classification accuracy.

In Table 2, we can see that the Keras Sequential API is used for the hierarchical structure of BdWood model layers. It begins with a convolutional layer that uses a 3 x 3 kernel and 64 filters. Non-linearity is introduced using the rectified linear unit (ReLU) activation function. Then, for spatial downsampling, a max-pooling layer with a 2 x 2 pool size is implemented. Batch normalization is then applied to improve stability during training. Two more convolutional layers follow, each with a greater number of filters (128 and 256, respectively), and they reproduce this convolutional-max-pooling-batch-normalization sequence iteratively. Layers are required for progressive abstraction and hierarchical feature extraction, which help the model identify complex patterns in the input data. After the convolutional layers a multi-dimensional tensor is converted into a one-dimensional vector by a flattening process. A fully connected layer with 512 neurons operated by the rectified linear unit (ReLU) receives this flattened representation as input. This completely connected layer introduces non-linearity, which helps to capture complex correlations in the data. A dropout layer with a dropout rate of 0.5 is intentionally introduced to reduce overfitting and guarantee strong generalization performance. CNN is the most popular deep learning algorithm that describes concepts, theory, and state-of-the-art architectures. The model architecture includes three convolutional layers followed by max-pooling and batch normalization layers to extract features from the images. A linear stack of layers. In CNN convolution layers used for feature extraction, the max-pooling layer reduces the dimensions of features. To stabilize and accelerate the training process, batch normalization is used to normalize the activation function of the previous layer. Converts the 2D output of the convolutional layers into a 1D vector, a flatten layer is used. Then, a dense layer is used as an output of a class score for each class. The algorithm of our proposed image classification technique, the Convolution Neural Network(CNN), is shown in Algorithm 1.

**Table 2:** Design choices.

List of Component	Design Choice
Kernel Size	(3, 3) for Conv2D layers
Filter Size	64, 128, 256 for Conv2D layers
Number of Layers	3 Conv2D layers followed by Dense layers
Dropout Rate	0.5 in Dense layer
MaxPooling	(2, 2) pooling after each Conv2D layer
Batch Normalization	After each Conv2D layer

### Feature explainability using Grad-CAM operation

Grad-CAM (Gradient-weighted Class Activation Mapping) is used to visualize which regions of an input image are the most important for a model. In our proposed convolutional neural network model, Grad-CAM helps to visualize the important regions that contribute to classifying our wood species in seven distinct classes. This explanation will provide a mathematical formulation of Grad-CAM.

Grad-CAM performs a global average pooling operation on the gradients to compute the importance weights $\alpha_{k}^{c}$ for each feature map ${\text{A}}^{\text{k}}$:

$$\alpha_{k}^{c}=\frac{1}{Z}\sum_{i,j}\frac{\partial y^{c}}{\partial A_{ij}^{k}}$$ (9)

Here, *Z* is the total number of spatial locations in the feature map ${\text{A}}^{\text{k}}$, and (*i*,*j*) denotes the spatial dimensions of the feature map and and ${\text{y}}^{\text{c}}$ is the prediction for class *c*.

The Grad-CAM heatmap is computed by performing a weighted combination of the feature maps ${\text{A}}^{\text{k}}$ using the importance weights $\alpha_{k}^{c}$:

$$L_{\text{Grad-CAM}}^{c}=\text{ReLU}\left(\sum_{k}\alpha_{k}^{c}A^{k}\right)$$ (10)

The ReLU function ensures that only the positive contributions are considered. ${\text{y}}^{\text{c}}$.

Finally, the heatmap $L_{\text{Grad-CAM}}^{c}$ can be upsampled to the size of the input image and visualized which parts of the image were most important.

**Algorithm 1. Customized CNN model (BdWood) for Bangladeshi indigenous wood species classification.**

1: **Initialize the Sequential Model:**

2: Create an instance of the Keras Sequential model to build the layer-by-layer structure of the CNN.

3: **Add First Convolutional Block:**

4:   Add a 2D Convolutional Layer with:

- Input shape of (224,224,3),
- 64 filters, each of size 3×3
- ReLU activation function.

5:   Add Max Pooling Layer with a 2×2 pool size to downsample spatial dimensions.

6:   Apply Batch Normalization to stabilize and accelerate training.

7: **Add Second Convolutional Block:**

8:   Add a 2D Convolutional Layer with:

- 128 filters, each of size 3×3
- ReLU activation function.

9:   Add Max Pooling Layer with a 2×2 pool size.

10:   Apply Batch Normalization.

11: **Add Third Convolutional Block:**

12:   Add a 2D Convolutional Layer with:

- 256 filters, each of size 3×3
- ReLU activation function.

13:   Add Max Pooling Layer with a 2×2 pool size.

14:   Apply Batch Normalization.

15: **Flatten:**

16: Convert the multi-dimensional tensor output from the previous layers into a 1-dimensional vector.

17: **Add Fully Connected Layer:**

18:   Add a Dense (fully connected) layer with:

- 512 neurons
- ReLU activation function.

19: **Add Dropout Layer:**

20:   Add a Dropout Layer with a rate of 0.5 to reduce overfitting.

21 **Output Layer:**

22:   Add a Dense layer with:

- Number of neurons equal to the number of classes (7 for seven wood species)
- Softmax activation function to output class probabilities.

Grad-CAM is a powerful tool for interpreting CNN models by highlighting the spatial regions that most affect the network’s prediction. The process involves computing gradients, pooling them to obtain importance weights, and generating a weighted combination of feature maps, followed by visualization.

Finally, all models are trained and tested by our newly developed indigenous wood species dataset and developed an android application. The algorithm of our proposed methodology is shown in Algorithm 2.

### Android application

Xylorix is an mobile app that provides automated wood identification through captured macroscopic end-grain images.

It’s work With just 3 simple steps: **1.** Cut-Snap-Identify, **2.** wood identification process becomes quick and simple, **3.** enabling faster processing and decision-making process in practical situation. Macroscopic image is the input image wood identification, it is the perfect companion for anyone in the field that needs to capture, record and share well-lighted macroscopic wood images.At the time it’s impossible to the general user to capture a macroscopic image https://www.xylorix.com/. Xylorix requires over hundreds of curated information and macroscopic end-grain images of wood species from around the world for referencing use, also provides a matching feature where users can identify wood species by comparing a live captured end-grain image of a wood with the images in the database. Meanwhile we proposed a simple and easy feature Android application that can used to classify the native wood species in Bangladesh.

**Algorithm 2. Wood species recognition using ensemble of deep learning and traditional ML models.**

**Require** List of pre-trained deep learning models (DenseNet121, ResNet152V2, Xception, VGG16, VGG19, MobileNetV2, InceptionV3), Custom CNN model (BdWood), Traditional ML models (KNN, SVM), Image dataset of wood species (WoodImages)

**Ensure** Classification results and accuracy for each input image   (BestModelPredictions)

1: **Step 1: Dataset Deployment**

2: Split WoodImages into training, validation, and test sets

3: **Step 2: Preprocessing**

4: **for** each image in WoodImages **do**

5:   PreprocessedImage ← resize(image, (224, 224))

6:   Add PreprocessedImage to PreprocessedImages

7: **end for**

8: **Step 3: Load Pre-trained Deep Learning Models**

9: SelectedModels ← [DenseNet121, ResNet152V2, Xception, VGG16, VGG19, MobileNetV2, InceptionV3]

10: LoadedModels ← loadModels(SelectedModels)

11: **Step 4: Train Custom CNN Model**

12: BdWood ← defineCustomModel()

13: Train BdWood on PreprocessedImages

14: **Step 5: Train Traditional ML Models**

15: Train KNN,SVM on PreprocessedImages

16: **Step 6: Generate Predictions**

17: **for** each image in PreprocessedImages **do**

18:   ModelPredictions ← []

19:   **for** each model in LoadedModels **do**

20:    Prediction ← PredictImage(model, image)

21:    ModelPredictions.append(Prediction)

22:   **end for**

23: **end for**

24: **Step 7: Ensemble Learning**

25: EnsemblePredictions ← []

26: **for** each prediction set in ModelPredictions **do**

27:   aggregatedPrediction ← Aggregator(prediction set)

28:   EnsemblePredictions.append(aggregatedPrediction)

29: **end for**

30: **Step 8: Model Selection and Evaluation**

31: AllModel ← [EnsemblePredictions + BdWoodPrediction + KNNSVMPredictions]

32: BestModel ← selectBestModel(AllModel, validation set)

33: Evaluate BestModel on test set

34: accuracy ← calculateAccuracy(BestModel, test set)

35: **Step 9: Explainability with Grad-CAM**

36: **for** each image in test set **do**

37:   GradCAMHeatmap ← generateGradCAM(BestModel, image)

38:   Display GradCAMHeatmap

39: **end for**

40: **Step 10: Android App Development**

41: Initialize Android app interface

42: **for** each uploaded image **do**

43:   UploadedImage ← preprocess(image)

44:   Prediction ← BestModel.predict(UploadedImage)

45:   Display Prediction on app interface

46: **end for**

47: **Output:** Display model accuracy, Grad-CAM visualizations, and Android app predictions

## Results

### Experimental setup

In our research, we used a newly collected native wood species dataset on different pretrained CNN image classification models and compared them with our proposed CNN models and an ensemble technique. In Table 3, we have shown that the dataset is labeled in seven different classes, and after applying the data preprocessing techniques, it is balanced; the instances increase and we used the instances for classification. All along a TensorFlow version 2.13.0 is used. Google Colab platform operates the entire code writing. The hardware is used such as 3.10 GHz to 3.11 GHz Intel Core i5-112300H processor, an NVIDIA Tesla K80 graphics card with Google Colab, and sixteen gigabytes of DDR5 RAM. Another hard- ware specification provided is a powerful 7-core GPU, 8 GB of RAM, 256 GB of storage, and an impressive 8-core M1 CPU.

**Table 3:** Classes of the dataset.

Name	Scientific	No. of	Augmented	Class
	Name	Image	Name	Label
Akashmoni	Acacia auriculiformis	359	1017	0
Eucalyptus	Eucalyptus globulus	296	1017	1
Kathal	Artocarpus heterophyllus	270	1017	2
Loha	Syzygium jambos	200	1017	3
Mehogoni	Swietenia mahagoni	257	1017	4
Minjiri	Senna siamea	245	1017	5
Raintree	Albizia procera	256	1017	6

This table is represented a quick overview of all classes in our datasets. This information is used to classify several CNN and ML models and choose the most suitable model for the given task based on its performance on both the Train and Test sets.

### Performance of traditional machine learning technique on an image dataset

**Fig 5:**
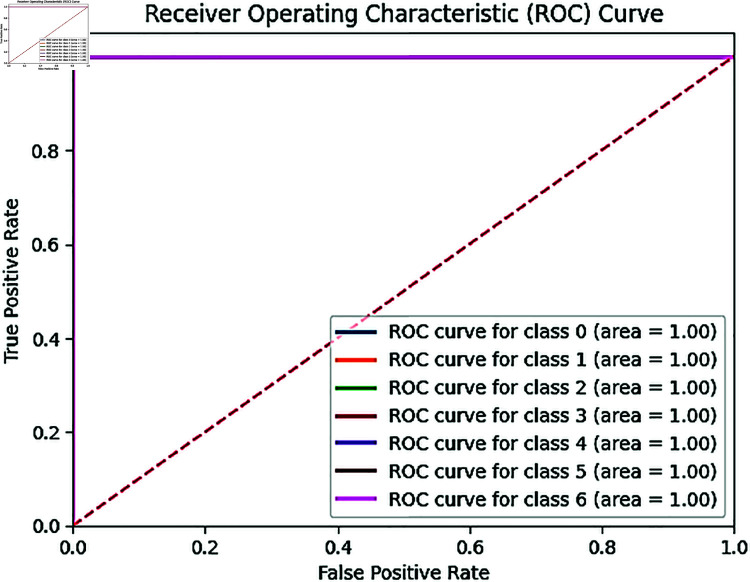
ROC curve for support vector machine.

**Fig 6:**
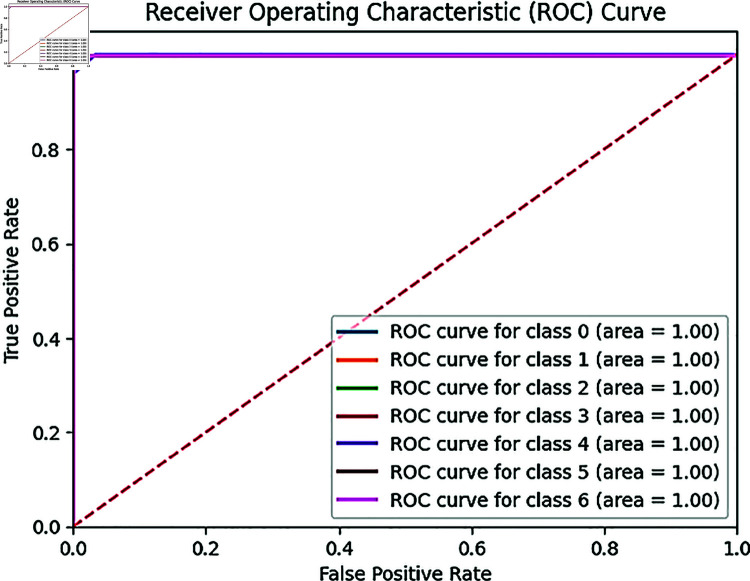
ROC curve for KNN where K = 3.

After applying traditional Machine learning algorithm on a wood species dataset, we achieved an identical accuracy score of 85.71% across all metrics for SVM and KNN is showed in Figs 5 and 6. This experimental result suggests us that the traditional SVM model perform consistently across the image classification. While similar to SVM, KNN achieved a little bit lower scores, with a slight edge in Precision (85.16%) compared to other metrics (85.14%).This difference indicates that KNN may misclassify slightly than SVM in some cases. Table 4 presents the performance metrics—Accuracy, F1 Score, Precision, and Recall—of the SVM and KNN classifiers.

**Table 4:** Performance measure of machine learning classifier.

Model	Accuracy	F1 Score	Precision	Recall
SVM	85.71%	85.71%	85.71%	85.71%
KNN	85.14%	85.14%	85.16%	85.14%

### Performance of pre-trained CNN models

At first, seven pre-trained CNN models were tested individually utilizing transfer learning techniques on our newly collected wood species dataset during the experiment, and the results of training and validation were carefully recorded. DenseNet121 performed brilliantly in the transfer learning scenario as well, with training accuracy of 97.09% and validation accuracy of 97.14%.

However,we see in Table 5 when it came to validation accuracy, InceptionV3, Xception, VGG19, MobileNetV2, and ResNet152V2 showed a generalization performance of about 93%. On the other hand, MobileNetV2 had the second lowest validation accuracy of 92.57%, while VGG16 performed the worst, with the lowest validation accuracy of 91.79%. During transfer learning, all seven of the models had excellent generalization and shared a lot of similarities. In terms of accuracy, DenseNet121, ResNet152V2 and VGG19, performed better than 95%. In this study, we evaluated our model with a great number of well-known deep learning models for a particular task and provided detailed performance metrics to evaluate the performance of each model’s effectiveness DenseNet121 demonstrated outstanding performance with a recall of 97.10%, precision of 97.29%, and an F1 score of 97.17%. With an F1 score of 94.03%, InceptionV3 showed excellent capabilities, while MobileNetV2 and VGG16 showed somewhat lower F1 values of 92.63% and 91.85%, respectively. With an F1 score of 93.75%, Xception showed efficacy, VGG19 achieved a commendable F1 score of 95.25%, and ResNet152V2 displayed robust performance with an F1 score of 95.45%.

**Table 5:** Comparative analysis for pre-trained models performance.

Model	Train Acc	Validation Acc	F1 Score	Precision	Recall
DenseNet121	97.09%	97.14%	97.17%	97.29%	97.09%
Xception	97.00%	93.64%	93.74%	93.87%	93.71%
ResNet152V2	96.82%	95.36%	95.45%	95.88%	95.30%
InceptionV3	95.70%	93.93%	94.03%	94.38%	93.91%
VGG16	91.41%	91.79%	91.85%	93.66%	91.62%
VGG19	92.16%	95.14%	95.24%	95.52%	95.13%
MobileNetV2	96.29%	92.57%	92.63%	92.81%	92.68%

Table 5 depicting the training, validation accuracy and all the performance matrices in individual training and testing the models. (Models: MobileNetV2, VGG19, VGG16, ResNet152V2, DenseNet121, InceptionV3, and Xception).

As we see in Table 6, the summary for each model represent the number of parameters, which are the weights and biases that the model learns during training. Model learns from the data by updating the trainable parameters. Total parameters are the sum of both trainable and non-trainable parameters. Weights and biases are the parameters that the model adjusts during training. It’s called trainable parameters. Non-trainable parameters come from layers like batch normalization and also fixed during the training phase.

**Table 6:** Pre-trained model parameters.

Model	Total Parameters	Trainable Parameters	Non-trainable Parameters
DenseNet121	7,037,504	6,953,856	83,648
Xception	20,861,480	20,806,952	54,528
ResNet152V2	58,331,648	58,187,904	143,744
InceptionV3	21,802,784	21,768,352	34,432
VGG16	14,714,688	14,714,688	0
VGG19	20,024,384	20,024,384	0
MobileNetV2	2,257,984	2,223,872	34,112

### Performance of our proposed multistage CNN model

In Table 7, we explain the break down of the layers and parameters of our CNN model to explain their roles and parameter counts.

**Table 7:** CNN model summary with parameters.

Layer (type)	Output Shape	Param #
conv2d (Conv2D)	(None, 222, 222, 64)	1792
maxpooling2d (MaxPooling2D)	(None, 111, 111, 64)	0
batch-normalization (BatchNormalization)	(None, 111, 111, 64)	256
conv2d_1_ (Conv2D)	(None, 109, 109, 128)	73856
max-pooling2d_1_ (MaxPoolin g2D)	(None, 54, 54, 128)	0
batch-normalization_1_ (BatchNormalization)	(None, 54, 54, 128)	512
conv2d_2_ (Conv2D)	(None, 52, 52, 256)	295168
max-pooling2d-2 (MaxPooling2D)	(None, 26, 26, 256)	0
batch-normalization_2_ (BatchNormalization)	(None, 26, 26, 256)	1024
flatten (Flatten)	(None, 173056)	0
dense (Dense)	(None, 512)	88605184
dropout (Dropout)	(None, 512)	0
dense_1_ (Dense)	(None, 7)	3591
Total params: 88981383 (339.44 MB)		
Trainable params: 88980487 (339.43 MB)		
Non-trainable params: 896 (3.50 KB)		

**Layer 1: Conv2D Layer (conv2d)**

Output shape: (None, 222, 222, 64)

Parameters: 1792

This is a 2D convolutional layer that applies 64 filters of size (3x3) to the input image. The 1792 parameters come from: 3x3 filters (for RGB input), so each filter has 27 parameters 27*X*64 = 1728 for 64 filters. 27*X*64 = 1728 weights. 64 bias terms, resulting in a total of 1728 + 64 = 1792 parameters.

Similarly we can calculate the parameters of all the layers in our proposed CNN model.

**Fig 7:**
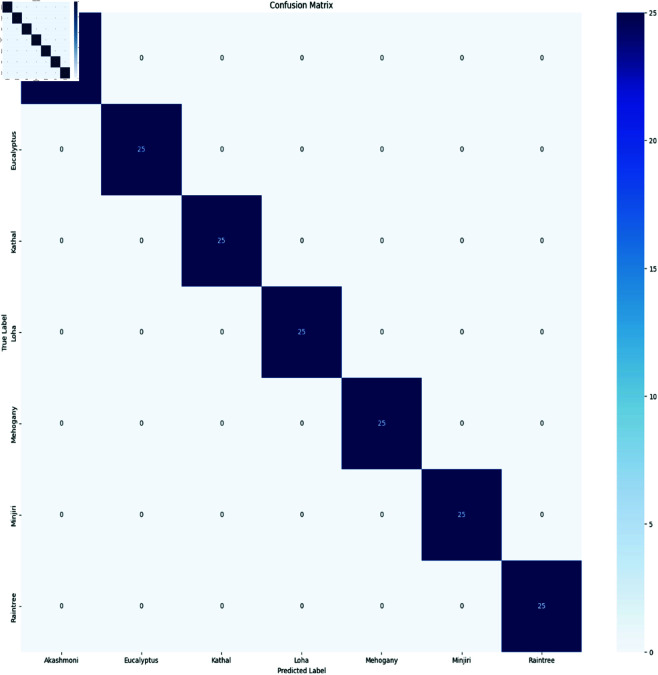
Confusion Matrixes for CNN model.

The confusion matrix works as a summary to describe the classification performance. we propose the confusion matrix for all models that we used for our classification. These confusion matrixes offer a united view of prediction outcomes, distinguishing correct and incorrect classification for our distinct seven classes. It compares the actual target values with the predicted values by the model. Each row of the matrix represents the actual class, while each column represents the predicted class. At first we discussed that, for n classes, the matrix becomes an NxN grid. In our customized Bdwood model, multiclass classifications is used for classifying the wood species, the diagonal elements represent the correct prediction and the off-diagonal elements represent the incorrect prediction in a confusion matrix. Fig 7, In our proposed classification models 25 instances of each class were correctly predicted for each class. No instances were incorrectly classified. We used a fixed batch size 32 with 0.0001 learning rate resulted in the highest accuracy.

**Fig 8:**
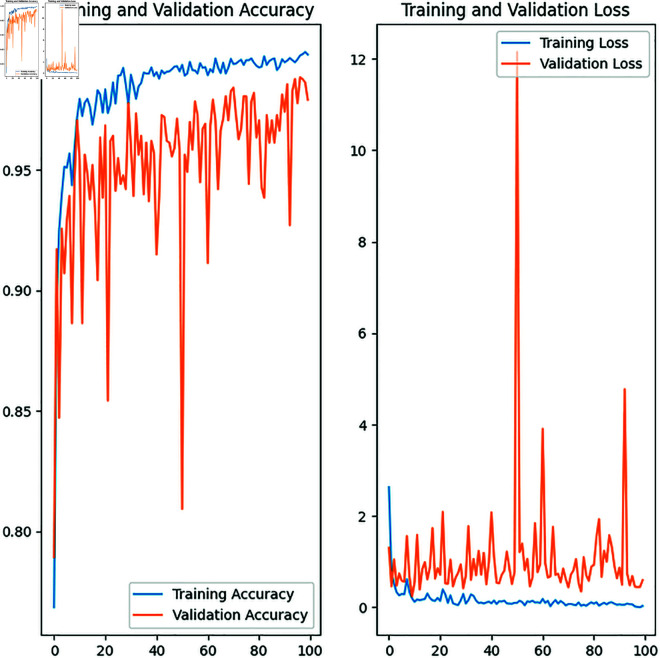
Loss curve for customized BdWood model.

From Table 8 In the early stages of training (epoch 1), validation and training losses are high. Specifies that our proposed model starts learning. The validation accuracy (78%) is slightly better than the training accuracy (76%), which indicating that the model is still in its early stages of adjusting the training and validation datasets. At epoch 20, the model improved significantly. The losses have dropped, and the training accuracy has increased to 97%. However, the validation loss is higher than the training loss, indicating that the model is learning well but could be slightly overfitting. Losses are extremely low (0.03). The training accuracy is 99% and the validation accuracy is 97%. This means that the validation loss has dropped to 0.59. So that we can understand, the model is continuing to improve. The difference between validation and training accuracy indicates that there is minimal overfitting. Reduction of validation loss indicates that the model is improving its generalization. Fig 8 shows that the model performance is strong after epoch 60, and further training provides diminishing returns.

**Table 8:** Model accuracy with different epoch.

Epoch	Validation loss	Loss	Validation accuracy	Accuracy
1	1.3	2.6	0.78	0.76
20	0.85	0.20	0.93	0.97
40	0.99	0.12	0.95	0.98
60	0.93	0.09	0.96	0.99
80	0.89	0.11	0.96	0.99
100	0.59	0.03	0.97	0.99

As we see in Table 9, Precision 97.94% measures that our proposed model is highly accurate in prediction. This precision tells us how well the proposed model is identifying the correct class when it makes a positive prediction. With 97.97% recall, our proposed model correctly identifying almost all positive instances, meaning there are very few false negatives (missed classifications). A 97.94% F1 Score means the model is well-balanced between precision and recall, making it robust for scenarios where false positives and false negatives are both important.

**Table 9:** Accuracy of CNN model.

Model	Train Acc	Validation Acc	F1 Score	Precision	Recall
**BdWood**	**99.80%**	**97.93%**	**97.94%**	**97.94%**	**97.97%**
**Model**					

The performance of our specially created model, which was painstakingly tailored for the intended objective, was equally impressive. With an F1 score of 97.94%, precision of 97.94%, and recall of 97.97%, this custom model demonstrated the benefits of customizing architecture for certain tasks.

**Fig 9:**
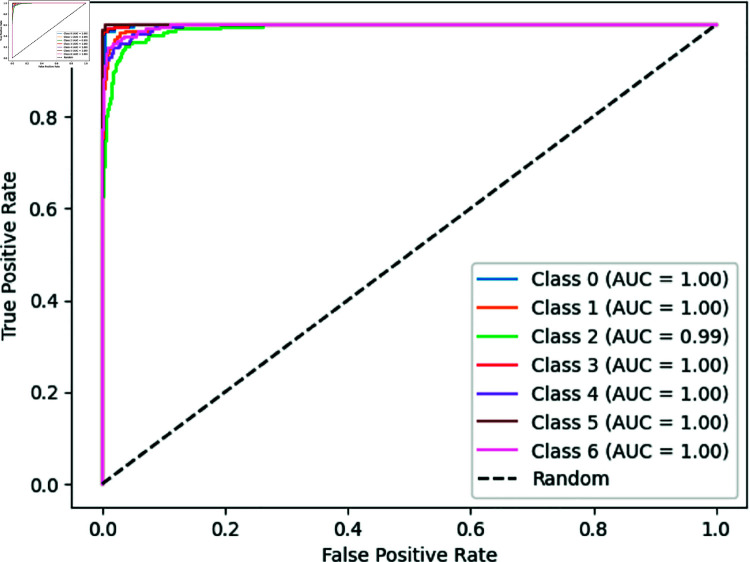
ROC-AUC curve for seven classes in convolutional neural network BdWood model.

In our experiment Fig 9 is shown, A ROC-AUC score of a customized BdWood CNN model is 99.85%, which means the model almost perfectly distinguishes between the sevens classes across all possible classification thresholds. Our proposed model is robust and performs well for applications. The performance is consistent across our newly collected wood species dataset and ROC-AUC of Bd wood model is a strong indicator of model excellence and effectiveness in distinguishing between classes. In practical conditions might introduce variations not captured during training.

**Fig 10:**
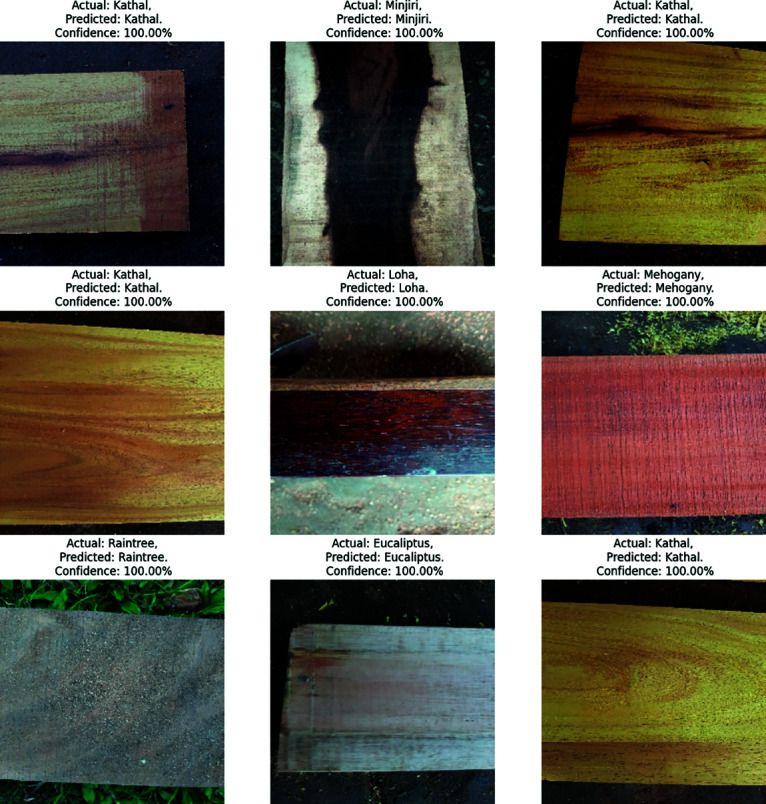
Prediction of all class using customized convolutional neural network BdWood model.

Our previous explanation we describe how important a feature for building a model for classification and prediction. Grad-CAM is a visualization technique used to highlight the important regions of an input image that a model required for making a prediction. It is applied on the high performed classification model to visualize the important features that are used to classify the model correctly and achieved a great accuracy. In ??, our proposed model shows the high accuracy for wood species classification. We have seven classes, using the Grad-CAM technique to highlight the important regions of the seven classes wood species. We see that the Grad-CAM technique can’t highlight the important region of the Loha class but other classes it seems better to highlight. Finally we explained our proposed model result where the actual class is Kathal, the predicted class is also Kathal with a confidence of 100%, meaning the ground truth is that this sample belongs to the “Kathal" class. The model also predicts “Kathal," which showed that it actually identified the sample. This means that the model assigned the highest probability (100%) to the “Kathal" class and 0% to the other six classes. In our classification when we applied the customized CNN model for wood species classification, we got 100% confidence for all the classes. Fig 10 is shown the above explanation.

An NVIDIA Tesla V100 GPU and 32 GB RAM is used for training a machine. Our proposed model is trained with 100 epochs with 30 minutes for the entire dataset. The training time per epoch was approximately 12 seconds and the total number of epochs was 100.For training, we use GPUs with at least 16 GB VRAM for ensuring efficient processing of large datasets. The training can also be performed on multi-GPU setups if larger datasets or deeper models are used. Table 10 presents the hyper parameters and settings, those are required used for compiling and training the model. Adam optimizer is selected for training, with a default learning rate of 0.0001. Sparse_categorical_crossentropy is a loss function, which is well-suited for multi-class classification problems. Accuracy is chosen as the evaluation metric to assess the model’s performance.

**Table 10:** Parameters list for model compile and fit.

List of Parameters	List of values
Type of optimizer	Adam optimizer
Type of learning_ rate	learning_rate=0.0001
Loss Function	sparse_categorical_crossentropy
Metrics	Accuracy
Epochs	100
Dropout	0.5

### Wood species model explainability with Grad-CAM

**Fig 11:**
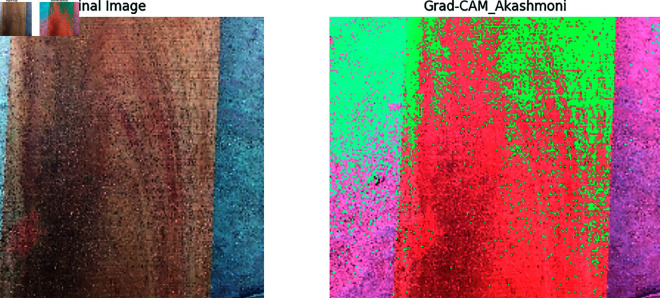
Gradcam result of class 0.

**Fig 12:**
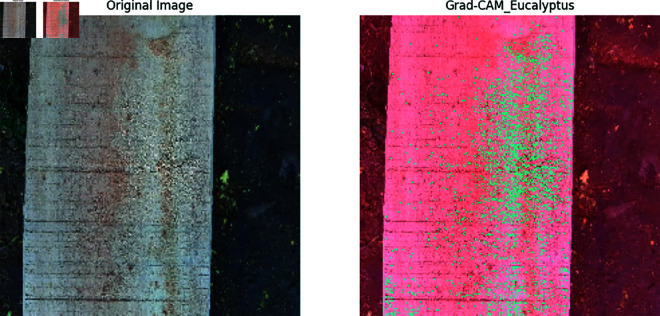
Gradcam result of class 1.

**Fig 13:**
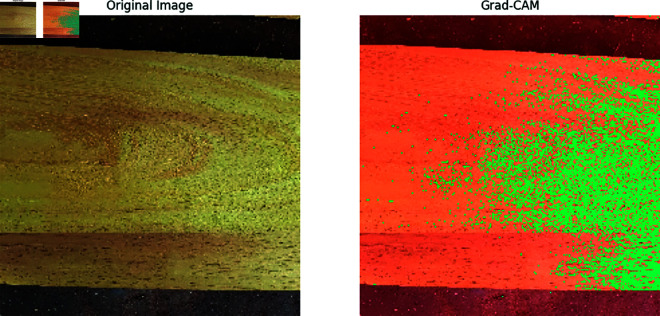
Gradcam result of class 2.

**Fig 14:**
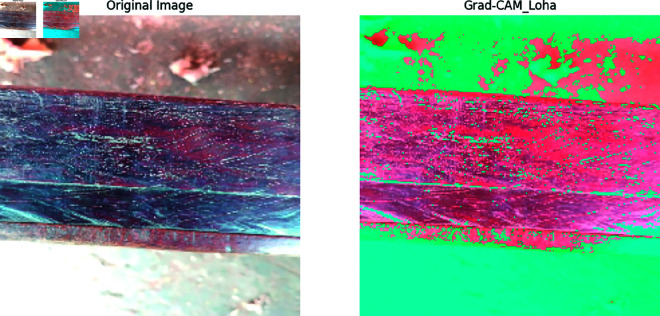
Gradcam result of class 3.

**Fig 15:**
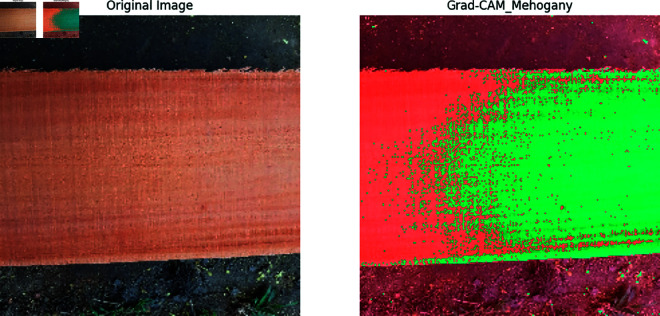
Gradcam result of class 4.

**Fig 16:**
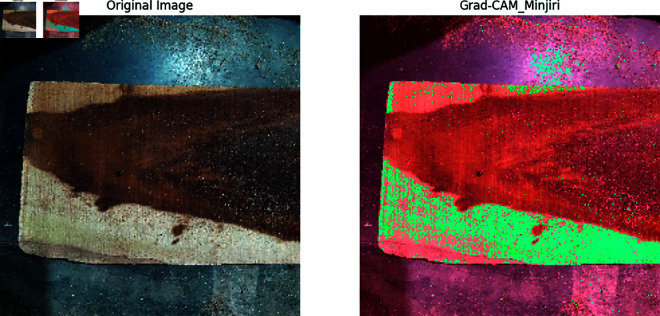
Gradcam result of class 5.

**Fig 17:**
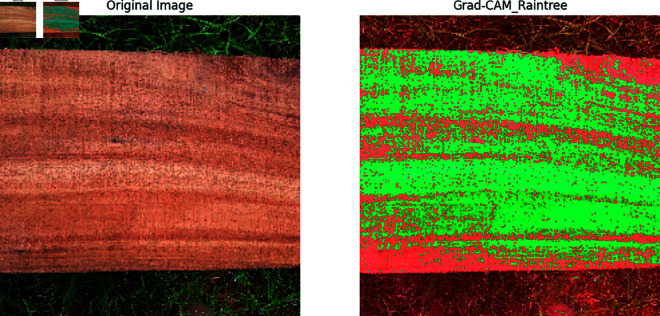
Gradcam result of class 6.

Our previous explanation we describe how important a feature for building a model for classification and prediction. Grad-CAM is a visualization technique used to highlight the important regions of an input image that a model requires to make a prediction. It is applied on high performed classification model to visualize the important features that are used to classify the model correctly and achieved a great accuracy. In Figs 11, 12, 13, 14, 15, 16, and 17, our proposed BdWood model shows the high accuracy for wood species classification. We have seven classes, using the Grad-CAM technique to highlight the important regions of the seven classes wood species.

**Red/Yellow Region analysis (High Activation):** These areas are the most important for the model decision. The model focused on these regions when classifying the image. For example, in your wood species classification task, if the red/yellow areas are on the texture or grain pattern, it means the model considers those features crucial for classification.

**Green/Blue Region Analysis (Moderate to Low Activation):** These regions were not the primary focus of the model for extracting the features that will help to classify the model. They may contain useful but less significant details.

**Dark/Black Region Analysis (No Activation):** The model found these regions irrelevant for the classification task. If a large portion of the image is dark, it means the model is ignoring that part, possibly due to background noise or uninformative features.

**Feature Explainability of Class 0,** the context of wood classification, this would be a clear image showing the wood surface of Class 0 (e.g Akashmoni wood in this case). Original image is the image that was provided as an input to the model. It represents the raw, unprocessed visual that the model was given to make a prediction. In Fig 11, Warm region red color specified the parts of the wood grain, texture, or color pattern that are the characteristic of Akashmoni wood. The model identified these unique features and used them to make its classification. If the warm regions are well-aligned with distinctive characteristics of Akashmoni wood, it indicates that the model learned the correct patterns during training. Green color region is the areas where the wood appears more uniform or featureless, such as the background or parts of the wood that don’t contribute to the classification.

**Feature Explainability of Class 1.** The context of wood classification, this would be a clear image showing the wood surface of Fig 12 (e.g Eucalyptus wood in this case).Very few region of this class is Green color and Green color regions had some influence but was not the primary focus of the model. They may contain useful but less significant details.

**Feature Explainability of Class 2.** A major contributor for classification is Red region areas which is the most important for the model’s decision. The model focused on these regions when classifying the image.The context of wood classification, this would be a clear image showing the wood surface of Class 2 (e.g Kathal wood in this case). Green color regions had less effect the model.

**Feature Explainability of Class 3.** In Fig 14, red region areas is the most important for the model’s decision. The model focused on these regions when classifying the image.The context of wood classification, this would be a clear image showing the wood surface of Class 3 (e.g Loha wood in this case). Green color regions had some influence but was not the primary focus of the model. They may contain useful but less significant details.

**Feature Explainability of Class 4.** In Fig 15, Mehogoni wood highlight with a green region in orginal image that feature was not appropriate features for classification. A small warm red region areas was created around the original image. This region is the most important for the model’s decision. The model focused on these regions when classifying the image.

**Feature Explainability of Class 5.** In Fig 16, A big black region exists in Minjiri orginal image that feature was unique for class 5. Warm Red region areas was created around this area. This region is the most important for the model’s decision. The model focused on these regions when classifying the image.

**Feature Explainability of Class 6.** In Fig 17, The highlighting result of the important region after applying Grad-CAM. The model is making its decision based on the important parts of the image. The wood’s grain and texture patterns are the good sign that can used for understanding the visual characteristics that define our wood dataset.

Finally, By analyzing the Grad-CAM outputs across multiple predictions, insights into whether the model is consistently focusing on correct or incorrect features. This can help in refining the dataset, model architecture, or training process.

### Applying cross-validation to BdWood model

Dataset is divided into multiple subsets for training and validation, the k-fold cross-validation technique is used to evaluate the performance of the Bdwood with seven newly collected wood species. Each sample is used for both training and testing as a subset instead of splitting a single data set into a training-test folder. The steps for cross-validation k-fold on the BdWood model split the data set into K-folds. The data set is divided into five subsets of equal sized. Each time using a different subset as the validation set while the remaining K-1 subsets are used for training. Then we have trained the model 5 times, and each time we divided the dataset in 5 different subsets, four for trained and one for validation set.

In each iteration, the BdWood CNN model is trained on K-1 folds and validated on the remaining fold. For example, in 5-fold cross-validation, if there are 7119 images, each fold contains 1017 images:

**1st iteration:** Train on Folds 1-4, validate in Fold 5.

**2nd iteration:** Train on Folds 1- 3,5, validate in Fold 4.

…

**5th iteration:** Train on Folds 2-5, validate on Fold 1.

Table 11 presents the hyper parameters and setting, that are required to compile and train the model. Adam optimizer is selected for training, with a default learning_rate of 0.001. For multiclass classification problems, categorical cross-entropy is well suited. Accuracy is chosen as the evaluation metric to assess performance. An early stopping mechanism is used to prevent overfitting. The model is trained with 100 epochs.The early stopping mechanism monitors the validation loss (val_loss) and stops training if there is no improvement for 10 consecutive epochs (patience = 10).Additionally, the restore_best_weights parameter is used to ensure that our model retains the weights from the best-performing epoch.

**Table 11:** Parameters list for model compile and fit.

List of parameters	List of values
Type of optimizer	Adam optimizer
Learning_rate	Default (00.001)
loss_function	Categorical_Crossentropy
Metrics	accuracy
Epochs	100
Callbacks	early_stopping
Restore Best Weights	True
Patience	10
Monitor	val_loss

Table 12 presents the result of the validation and training accuracy for each fold during cross-validation. We split the data into five different folds. Each fold consists of seven wood species classes with 205 wood image for each class. It helps to assess the model’s generalization ability. Fold 2 is shown with the highest validation accuracy (98.61%), while the lowest occurs in Fold 3 (91.29%), It will be suggested that the model performance varies across different fold splits. Similarly, the training accuracy ranges from 89.81% (Fold 3) to 98.72% (Fold 2). **Early Stopping:** we implemented early stopping to halt training when the validation loss stops improving for a certain number of epochs (patience = 10). This helps to prevent overfitting during trained the model. **Dropout:** We applied dropout 0.5 for random drop neurons during training, thus reducing the risk of overfitting.

**Table 12:** Validation and training accuracy across folds.

Validation Folder	Validation Accuracy (%)	Training Accuracy (%)
Fold 1	97.56	96.38
Fold 2	98.61	98.72
Fold 3	91.29	89.81
Fold 4	94.43	94.43
Fold 5	96.43	96.43
Average	95.46	95.15

### Performance of ensemble technique

**Fig 18:**
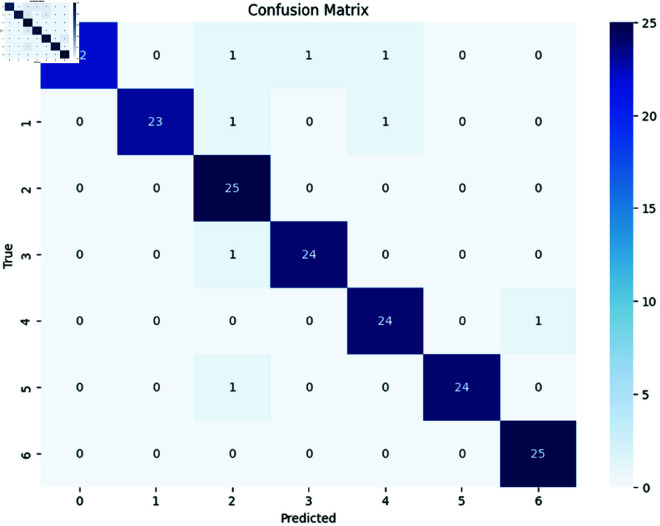
Confusion matrix for ensemble technique.

Ensemble technique bagging was applied only one epoch for training the seven pretrained models in our experiment that must be benefited than individual model training. Moreover, each model in the ensemble is fine-tuned across several iterations on the training data. In our experiment every model learns and adjust their individual accuracy typically improves over 100 epochs. At the same time in Table 13, the ensemble bagging technique shows a great accuracy around 95% over 1 epoch in our newly collected wood species datasets. Fig 18 presents the confusion matrix of the proposed ensemble model, illustrating its classification performance across all classes. The diagonal elements represent correctly classified instances, while the off-diagonal elements indicate misclassifications. A higher concentration of values along the diagonal suggests strong predictive accuracy.

**Table 13:** Classification report for ensemble technique.

Class level	Precision	Recall	F1-score	Support
Class 0	1.00	0.88	0.94	25
Class 1	1.00	0.92	0.96	25
Class 2	0.86	1.00	0.93	25
Class 3	0.96	0.96	0.96	25
Class 4	0.92	0.96	0.94	25
Class 5	1.00	0.96	0.98	25
Class 6	0.96	1.00	0.98	25
**Accuracy**	1.00 (175 samples)			
**Macro average**	0.96	0.95	0.95	175
**Weighted average**	0.96	0.95	0.95	175

**Fig 19:**
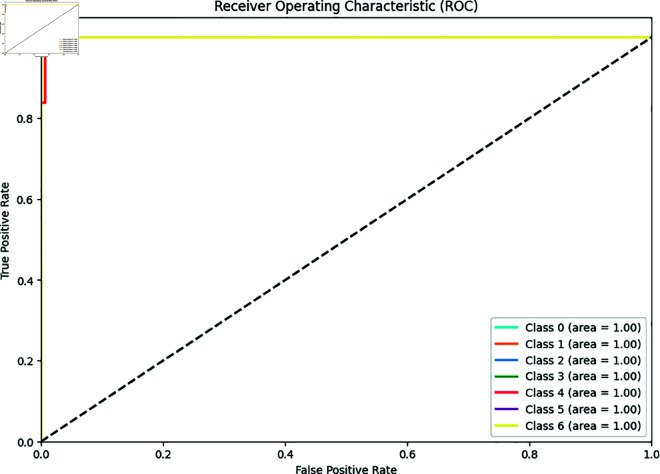
ROC curve for ensemble technique.

Fig 19 is shown, a ROC-AUC score 1.00 depicts that our heterogeneous ensemble technique shows a perfect classification for wood species classification in seven different wood species in northern Bangladesh. It’s demonstrating the perfect discrimination between the classes.

### Performance comparison

In Tables 14 and 15, the comparative analysis of traditional machine learning, pretrained CNN, sequential CNN, ensemble bagging models focus on accuracy and loss across training, validation, and test datasets. In our proposed model the lowest training loss (3.32%) shows a better optimization process and perfect learning from the training dataset. Our proposed model indicates a perfect performance across 100% test accuracy with 0% loss. In light of our result, our proposed model testing loss is less than other, and it shows great accuracy as well.

**Table 14:** Comparative result analysis (Accuracy).

Model	Epoch	Loss	Train	Validation	Validation	Test	Test
		values	Accuracy	Loss	Accuracy	Loss	Accuracy
**BdWood**	**100**	**3.32%**	**99.80%**	**59.68%**	**97.93%**	**0.00%**	**100%**
DenseNet121	100	8.66%	97.09%	8.76%	97.14%	0.30%	100%
Xception	100	8.57%	97.00%	19.98%	93.64%	3.10%	98.86%
ResNet152V2	50	9.10%	96.82%	17.32%	95.36%	6.00%	99.43%
InceptionV3	100	12.66%	95.70%	18.90%	93.93%	5.56%	97.14%
VGG16	100	24.08%	91.41%	20.92%	91.79%	8.16%	97.71%
VGG19	100	21.71%	92.16%	13.28%	95.14%	2.69%	98.86%
MobileNetV2	50	11.08%	96.29%	24.71%	92.57%	4.99%	98.86%
Ensemble	1	-	-	-	-	-	95.43%
SVM	-	-	-	-	-	-	85.71%
KNN	-	-	-	-	-	-	85.14%

**Table 15:** Comparative result analysis of performance Matrix.

Model	F1 Score	Precision	Recall	ROC-AUC
**BdWood**	**97.94%**	**97.94%**	**97.97%**	**99.85%**
DenseNet121	97.17%	97.29%	97.09%	99.93%
Xception	93.74%	93.87%	93.71%	99.68%
ResNet152V2	95.45%	95.88%	95.30%	99.80%
InceptionV3	94.03%	94.38%	93.91%	99.69%
VGG16	91.85%	93.66%	91.62%	99.67%
VGG19	95.24%	95.52%	95.13%	99.84%
MobileNetV2	92.63%	92.81%	92.68%	99.48%
Ensemble	96.00%	95.00%	95.00%	100%
SVM	85.71%	85.71%	85.71%	99.93%
KNN	85.14%	85.16%	85.14%	99.93%

**Fig 20:**
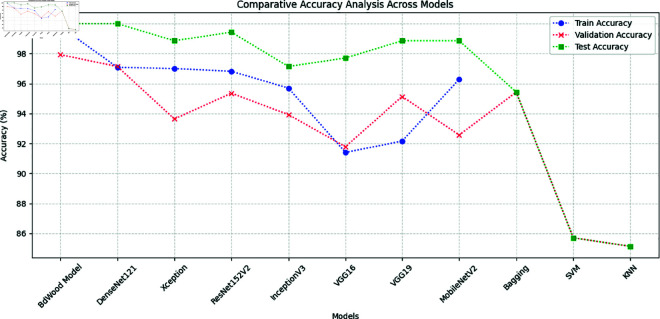
Overview of the ML, CNN, Customized BdWood, Ensemble models accuracy.

During the last phase of our analysis, the comparative results are displayed in Fig 20. Here, we compared the the result of Pre-trained CNN models, ensemble bagging technique and traditional Machine Learning with our proposed model performance matrices.

**Fig 21:**
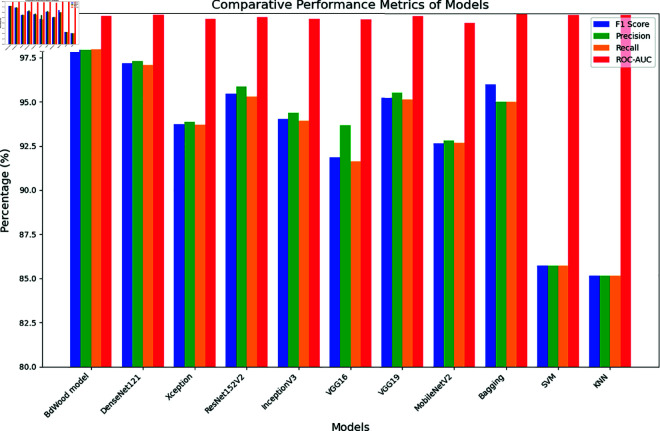
Overview of precision, f1 score, recall and ROC-AUC curve.

As we see in Fig 21, F1, precision, recall, and ROC values for all models are depicted on the condition that these model is executed with a great accuracy and also classified our seven class accurately.

Among all these models, the CNN image classification model outperforms. Most traditional machine learning and pre-trained models concur with our customized model in this research. This approach is designed to speed up image data processing. These results manifest the potential of the proposed framework.

Traditional machine learning and ensemble bagging technique shows lower performance that reveal less accuracy in generalization

Some similarities can be identified among these models, namely:

- All models have AUC scores above 0.99, it seems a great classification abilities.
- Traditional ML models, Pre-trained CNN models, ensemble technique and our proposed model depict strong performance.
- F1 score, Precision, and recall all exceed 0.92 for CNN models. A precision above 0.92 means that all the models predictions are 92% correct. This recall above indicates that 92% of the actual positive cases. showing its effectiveness in capturing most of the relevant instances. With an F1 score means that the balance between precision and recall is strong.

Overall, these metrics demonstrate that the model effectively detects and classifies the target cases with high accuracy.

Some differences may also be discerned. These are:

- Across these key metrics scores range from 0.97 (Proposed CNN model) to 0.85(traditional Machine learning), representing the divergent of effectiveness.
- Our proposed BdWood model, a Customized technique, outperforms standalone models.

Grasping these nuances can help inform the selection of a model tailored for specific classification tasks in wood species classification.

Consequently, the DenseNet121 model proves to be a valuable approach for identifying Bangladeshi wood species, complemented by the use of our customized BdWood model to enhance accuracy. In practical scenarios, these models can be employed to accurately identify Bangladeshi timber, thereby reducing the occurrence of accidents.

## Performance of android application

Android’s development environment are written primarily in Java or Kotlin. Android applications allow users to perform a variety of tasks. Android represent the user interface (UI) screens of the app. Each screen is usually represented by an activity, which manages user interactions with the app. In Table 16 is shown the image Classification of wood species under different Lighting Conditions using An Android application.

**Table 16:** Performance evaluation under different lighting conditions.

Total Images	Correctly Identified	Misclassified Images	Lighting Condition
100	85	15	Bright Daylight
100	78	22	Dim Indoor Light
100	95	5	Natural Shade
100	82	18	Artificial Light

**Fig 22:**
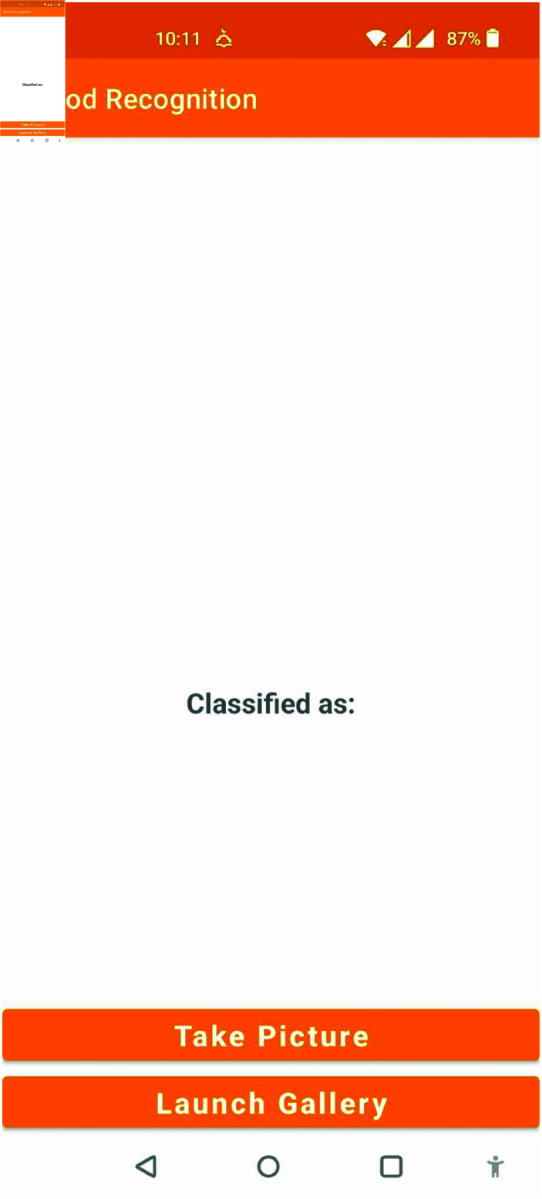
Input image from some input storage.

**Fig 23:**
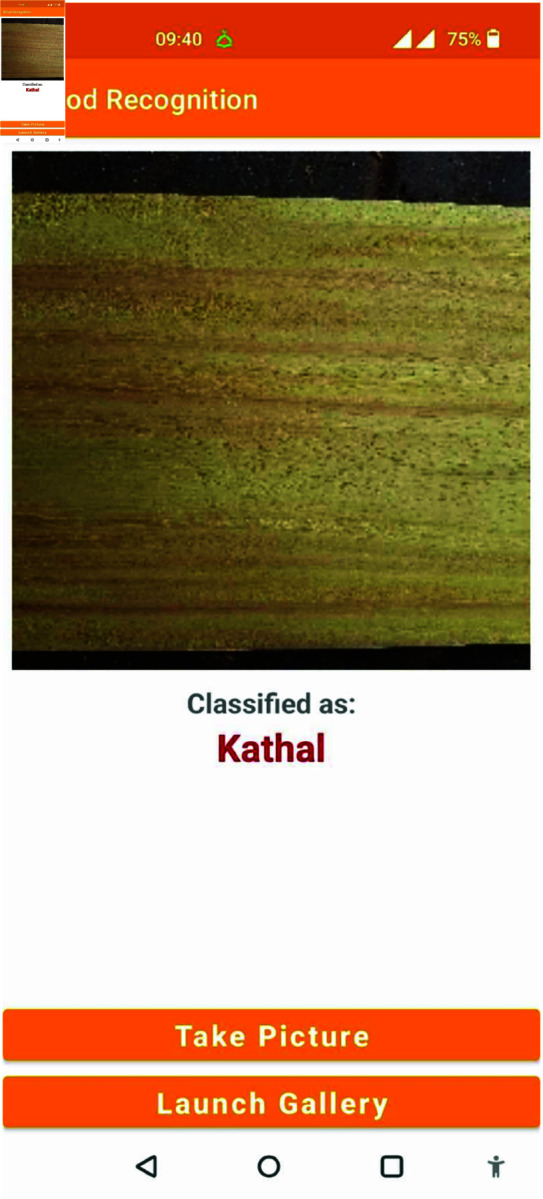
Result of the App prediction.

An Android application is a great tool to represent our proposed classification model that enables users to perform wood species identification on their Android devices. Using our predicted result we applied the best accuracy model to build an Android app. It consists of two feature to capture an image or import image from gallery that can be used for classification and shown Fig 22 and 23, the class name of wood specie.

## State-of-the-art analysis for wood dataset and wood species classification

Various factors such as the data sets, model architecture, and data pre-processing techniques are required to measure the performance and the accuracy of ML and DL models for wood image classification. This section explains the comparison between Bdwood model and the performance of existing works. In recent years, various research have focused on the wood species classification using various datasets. Our proposed Bdwood model performance is evaluated against previous approaches, considering the models applied and their accuracy. Tables 17 and 18 explain that the proposed approach performs outstanding than the existing approaches of wood species classification using our newly collected indigenous wood species dataset.

**Table 17:** Comparative analysis of image classification techniques.

References	Dataset Details	No of Image	Classifier	Accuracy
[38]	Stereo	2390	ANN	89.6
[39]	Micro	2240	SVM	55.3
[18]	XCT	240	k-NN	98.3
[40]	Micro	2240	SVM	80.7
[41]	Macro	2942	SVM	56.0
[42]	Micro	2240	SVM	66.3
[43]	Micro	1500	SVM	79.9
[44]	Micro	1221	k-NN (k=1)	85.0

**Table 18:** Comparison of CNN-based wood classification studies.

Study	Dataset	Image Type	Specie	Model	Accuracy
		Type	Images	Architecture	%
[21]	UFPR (Macro)	Macro	41 / 2942	3-ConvNet	95.8
[21]	UFPR (Micro)	Micro	112 / 2240	3-ConvNet	97.3
[45]	Softwoods	Macro	5 / 16,865	LeNet	99.3
[46]	Softwoods	Macro	5 / 33,815	Ensemble	98.0
[34]	Meliaceae Species	Stereomicro	10 / 2303	VGG16	88.7 (c)
[47]	FRIM Collection	Stereomicro	100 / 101,446	SqueezeNet	77.5
[48]	FWRC Collection	Stereomicro	10 / 1869	InceptionV4 + ResNetV2	92.6
[49]	UFPR (Micro)	Micro	112 / 2240	ResNet101	96.4
[50]	Brazilian Species	Stereomicro	281	DenseNet	98.8
[6]	Meliaceae Species	Stereomicro	10	ResNet34	81.9, 96.1
[51]	European Species	Macro	14 / 312	Residual Convolutional	98.7

Table notes: This table compares the performance of CNN-based wood classification models across various studies, datasets, and image types. Accuracy values reflect the respective models’ performance on different datasets.

Our proposed BdWood model achieves an impressive ROC-AUC score of 99.85%. This outstanding value demonstrates our proposed Bdwood model differentiate between the seven wood species classes in Bangladesh. Both positive and negative samples are correctly classified using our proposed Bdwood model with minimal overlap. Compared with other existing models, including Ravindran *et al*. [34], which reported ROC-AUC values ranging between 88.7% to 97.5%, and Ravindran [6], who achieved ROC-AUCs of 81.9% and 96.1% on Meliaceae species datasets. Finally, Bdwood model provides high accuracy, balanced F1 score, Precision, and Recall, and its near-perfect ROC-AUC score so that we can classify the seven classes with a newly collected image datasets of wood species.

The BdWood model performs outstanding when we compare with the CNN-based wood species classification models with not only due to its high accuracy but also due to its balanced performance across multiple metrics. The model’s results are in the top tier when compared with others such as:

- Hafemann *et al*. [21] with an accuracy of 95.8% (Macro) and 97.3% (Micro) using 3-ConvNet.
- Lens *et al*. [49] with 96.4% accuracy using ResNet101.
- Tang *et al*. [47] with a slightly lower accuracy of 77.5% using SqueezeNet, showcasing BdWood model superiority in terms of robustness and classification power across diverse datasets.

## Discussion

In line with our aims, we evaluated our proposed classification method data balancing technique against seven pretrained CNN models, ensemble technique and two traditional machine learning classification methods, where our proposed Bdwood model performed outstanding to others. Among all classifiers tested, customized Bdwood model achieve an F1-score of 97.94%, Precision of 97.94%, Recall of 97.97%, ROC of 99.85% and test accuracy of 100%. After that, we applied a feature highlighting technique Grad-CAM into our best performed BdWood model that can highlight the important region required for our classification. This technique emphasized our newly collected wood species features, these features were required for classification.While our proposed model demonstrates high performance on the newly developed dataset of seven Bangladeshi wood species, we have not apply our proposed model on a broader datasets or wood species of different geographical regions. Variations in environmental factors, growth conditions, and dataset characteristics could impact model performance. We will explore transfer learning techniques to adapt our proposed multi-stage CNN model to a broad datasets of different region wood species that investigation can enhance its generalizability. This generalizability directly addresses the concern and provides actionable future research directions. Finally, we developed and Android application for identifying the seven indigenous wood species in Bangladesh. This research seeks to develop a model that delivers accurate predictions while also developing an application that can be used by the general people for correct species selection, and supporting the conservation of threatened species.

## Conclusion

In conclusion, our research focuses the critical challenge of collecting indigenous wood species as a brand-new dataset, models are trained on fresh, unseen data. The performance of various pre-trained deep learning models demonstrated high accuracy, with DenseNet121 achieving the best results. Moreover, we proposed a BdWood model outperformed all the existing techniques. Traditional machine learning algorithms such as KNN and SVM were compared against deep learning approaches. After that, we applied Grad-CAM, highlighting the important features of the wood species, ensuring that our collected datasets are applicable for ML and CNN classifiers. Our comparative study on an heterogeneous ensemble technique and a Sequential CNN model are also shown the accurate classification results. The practical contribution of our work has developed an android application, that can be used in real-world scenarios for wood identification. Finally, our research advances the field of model interpretability and deep learning-based image classification in novel, real-world datasets.
